# Diagnosis on Excision Biopsy of Breast Tissues Labeled As Fibroepithelial Tumors on Trucut Samples in a Developing Country

**DOI:** 10.7759/cureus.18111

**Published:** 2021-09-19

**Authors:** Maliha Latif, Asif Loya, Maryam Hameed, Usman Hassan, Sajid Mushtaq, Mudassar Hussain

**Affiliations:** 1 Histopathology, Shaukat Khanum Memorial Cancer Hospital and Research Centre, Lahore, PAK; 2 Pathology, Shaukat Khanum Memorial Cancer Hospital and Research Centre, Lahore, PAK

**Keywords:** diagnosis, histopathology, excision biopsy, fibroepithelial lesion, trucut biopsy

## Abstract

Introduction: Fibroadenomas (FAs) and phyllodes tumors (PTs) are less prevalent but allied to have malignant transformation in many instances. It is a challenge to diagnose the phyllodes by conventional trucut biopsy technique.

Objective: To evaluate the histological characteristics of tumors labeled as fibroepithelial lesions of breast tissues on trucut biopsy and compare with a diagnosis on excision biopsy.

Methods and materials: It was a descriptive cross-sectional study that was carried out in Shaukat Khanum Memorial Hospital and Research Centre within six years from January 2015 to January 2021. In trucut samples, stromal cellularity, stromal cell nuclear atypia, mitotic count, stromal overgrowth, the enhancement of stromal cellularity adjacent to epithelium were scrutinized. In each category, the activity was seen as absent, mild, moderate, or severe. Mitotic activity was graded as 0-1, 0-5, 5-10, >10.

Results: A total of 125 patients were registered for the study. The mean age of patients in our study was 33.86 ± 9.95 years. The mean size was 41.02 ± 27.38 mm with a mean lump duration of 7.52 ± 5.34 months. In the FA group, the trucut sampling report showed the stromal cellularity as mild in 62 (69.7%) and stromal cell nuclear atypia as absent in 68 (76.4%) cases. But in the phyllodes tumor group, the stromal cellularity was severe in 10 (27.8%) patients and stromal cell nuclear atypia was severe in five (13.9%). The ultimate outcome showed that 89 (71.2%) patients had FA and 36 (28.8%) had PT at excision.

Conclusion: Assessment of tumor size, stromal cellularity, mitoses, and enhancement of stromal cellularity adjacent to epithelium are useful markers for diagnosing the PT in trucut needle biopsy.

## Introduction

In women, breast cancer is the most common of all tumors [1]. Breast fibroepithelial tumors are a type of biphasic neoplasm that fallouts from the development of both stromal and epithelial components, and include cellular fibroadenoma (FA), FA with cellular stroma, fibroepithelial lesion with cellular stroma, phyllodes tumor (PT), or cyst sarcoma phyllodes [2] They are prevalent in young women and middle-aged women's and are allied to increase the incidence of malignant transformation in many instances [3].

PTs are uncommon fibroepithelial tumors that are cataloged as benign, borderline, and malignant histologically. Regardless of the fact that only around 10% of these tumors are malignant, even benign tumors may increase in size and relapse. Because of their proclivity for local recurrence, patients are generally treated by surgical excision with negative surgical margins [4,5].

An FA is a painless, firm, mobile breast lump that frequently affects women between 20 and 35 years. Bilateral FAs and multiple FAs are frequently reported in many cases. Screening mammography could detect the nonpalpable FAs and sclerosis with “popcorn” calcifications. Ultrasound assists in detecting and portraying FAs precisely [6,7].

In many institutions, Excision biopsy is preferred over trucut biopsy for phyllodes [8]. A significant advantage of this strategy is that pathologists acquire adequate tissue for yielding a definite diagnosis. It helps physicians and surgeons in making an appropriate treatment and management plan [9,10].

Despite the use of numerous diagnostic markers, the differential diagnosis of fibroepithelial tissues with trucut biopsy is sometimes challenging and imprecise. And it happens particularly due to the heterogeneity of the stromal component and enhanced cellularity of tumors [11]. So, pathologists label these tissues as “fibroepithelial lesions.” Surgical excision is advised for tumors more than 30 mm in size with increased mitosis and further histological scrutiny is recommended for further clarification [12].

Regarding the management of patients, it is either observation and monitoring of the size of the tumor over a period of time or excision at a given moment. So, it is crucial to determine the reliable markers on trucut biopsy, that could help in understanding the benign or malignant nature of an FA. The aim of our study was to evaluate the clinical and histological characteristics of tumors that were labeled as fibroepithelial lesions after trucut tissue biopsy and comparing with diagnosis based on excision biopsy.

## Materials and methods

It was a descriptive cross-sectional study, carried out in Shaukat Khanum Memorial Hospital and Research Centre from January 2015 to January 2021. We included all the patients who underwent surgical excision of breast lesions after trucut biopsy result of a fibroepithelial tumor after IRB approval of Shaukat Khanum memorial cancer hospital and research center (SKMCH &RC) IRB # EX-30-12-19-02. Patients with no excisional biopsy were excluded from the study.

Histopathological evaluation was performed by two pathologists of the hospital in the department of histopathology. On trucut biopsy samples, we executed the activities of the stromal cellularity, stromal cell nuclear atypia, mitotic count, stromal overgrowth, the enhancement of stromal cellularity adjacent to the epithelium in all samples. In each category, these (above mentioned) activities were seen as absent, mild, moderate, or severe. Mitotic activity was graded as 0-1, 5-10, >10. While in the enhancement of stromal cellularity adjacent to the epithelium, we recognized the absence, not applicable, or the presence of activity. “Not applicable” applied to cases in which the trucut core was not sufficient to differentiate the epithelium and stromal activity. In excision samples, FA and phyllodes were diagnosed based on specific markers. We strained to identify the parameters in trucut biopsy, which were corresponding to the diagnosis based on the excisional biopsy.

Each category was graded based on peculiar histopathological features. For stromal cellularity, most cellular area of the slide was assessed. Approximately twice the cellularity of that of normal perilobular stroma, with none or rare stromal nuclei, was labeled as mild and within close contiguity with many stromal cells, nuclei were labeled as Marked stromal cellularity. The intermediate degree was between mild and marked with cellularity more than twice that of normal perilobular stroma and scattered stromal nuclei.

Stromal cell nuclear atypia was graded as 1 (mild), 2 (moderate), or 3 (marked). Stromal cells with grade 1 nuclei have small, uniform nuclei, with evenly dispersed chromatin. Grade 2 stromal nuclei were moderately increased in size, with occasional nucleoli and grade 3 nuclei much variable in nuclear size and shape, with irregular nuclear Membranes. Stromal cell mitotic figures were counted in the most mitotically active areas and expressed as per mm^2^. Stromal overgrowth was defined as the presence of stroma without epithelium in any low-power field to high power field (×40). The relative proportion of stroma to the epithelium was estimated semi-quantitatively as the overall relative percentage of stroma compared with epithelium present in at least three representative low-power fields [3,13].

Mean with standard deviation were calculated for continuous variables like age of the patient, size of the tumor, duration of clinical presentation. Categorical data like a side of the tumor, location of the tumor, specific surgical procedure, ultrasonographic characteristics, and histopathologic results from trucut biopsy and excisional biopsy were analyzed with frequency and percentages. The outcome was confirmed as FA or PT on excisional biopsy. The Chi-square test was also used to check the significant difference at p-value (p < 0.05). Statistical analysis was performed with SPSS 23 version (SPSS Inc., Chicago, IL).

## Results

We enrolled a total of 125 patients. The mean age of patients was 33.86 ± 9.95 years. Fifty patients (40.0%) had lump in left breast, while 75 (60.0%) on right side. The mean size was 41.02 ± 27.38 mm with a mean lump duration of 7.52 ± 5.34 months. The mammographic findings showed a circumscribed hypoechoic lobulated mass in 57.2%, heterogeneously hypoechoic mass with lobulated margin in 30.8%, and irregular hypoechoic mass in 12.0% (Table 1).

**Table 1:** Demographic and clinical variables

Variables	Results
Age (Years)	33.86 + 9.95
Lump in Left breast: Lump in Right breast	50 (40.0%): 75 (60.0%)
Duration (Months)	7.52 + 5.34
Size (mm)	41.02 + 27.38
Outcome; Fibroadenoma at excision: Phyllodes tumor at excision	89 (71.2%): 36 (28.8%)

Regarding the percentage of tumors with relation to ultrasonographic size and excisional biopsy diagnosis, we found that FA was less than 30 mm in 44 (49%) cases, while more than 30 mm in 45 (51%). While regarding phyllodes, only five (14%) were less than 30 mm, and 31 (86%) were found to have an ultrasonographic size of more than 30 mm (Table 2).

**Table 2:** Percentage of tumors with relation to ultrasonographic size at time of excision

Tumor size on ultrasound	Fibroadenoma at excision (n=89)	Phyllodes tumors at excision (n=36)	Total	P-value
Less than 30 mm	44 (49%)	05 (14%)	49 (39%)	0.000
More than 30 mm	45 (51%)	31 (86%)	76 (61%)	
Less than 20 mm	06 (6.7%)	01 (2.7%)	07 (5.6%)	0.346
More than 20 mm	83 (93.3%)	35 (97.3%)	118 (94.4%)	

Regarding the procedure types, 84 (67.2%) patients underwent a lumpectomy, mastectomy in five (4.0%), nipple-sparing central segmentectomy in one (0.8%), nipple-sparing segmentectomy in one (0.8%), segmentectomy was performed in four (3.2%), wide local excision was performed in 26 (20.8%), and wire localized lumpectomy was performed in four (3.2%) patients.

The outcome showed that 89 (71.2%) patients had FA at excision and 36 (28.8%) patients had PTs. The final histology also revealed the different types of FA and PT on excisional biopsy.

We found that in trucut specimens of patients who were finally diagnosed with FA, the stromal cellularity was mild in 62 (69.7%), moderate in 27 (30.3%), and no severe stromal cellularity. While, in PT patients, the stromal cellularity was mild in nine (25.0%), moderate in 17 (47.2%), and severe in 10 (27.8%) patients.

Regarding the stromal cell nuclear atypia on trucut specimens, it was absent in 68 (76.4%) and mild in 21 (23.6%), but moderate or severe atypia was absent. Similarly, in PTs, the stromal cell nuclear atypia was absent in 30 (30.6%), mild in 15 (41.7%), moderate in five (13.9%), and severe in five (13.9%) patients.

Mitotic count was 0-1 in 89 (100%) patients of FA. While in PT, the mitotic count was found 0-1 in 18 (50.0%) patients, 1-5 in six (16.7%), 5-10 in five (13.9%), >10 in seven (19.4%) patients (Table 3).

**Table 3:** Histologic features of fibroepithelial lesions on trucut samples in comparison to diagnosis on excisional biopsy

Histologic features on trucut biopsy	Diagnosis at excision			P-value
	Fibroadenoma (n=89)		Phyllodes tumor (n = 36)	
Stromal cellularity				
Mild	62 (69.7%)	9 (25.0%)		0.001*
Moderate	27 (30.3%)	17 (47.2%)		
Severe	0 (0.0%)	10 (27.8%)		
Stromal cell nuclear atypia				
Absent	68 (76.4%)	30 (30.6%)		0.000*
Mild	21 (23.6%)	15 (41.7%)		
Moderate	0 (0.0%)	5 (13.9%)		
Severe	0 (0.0%)	5 (13.9%)		
Mitotic Count				
0-1	89 (100.0%)	18 (50%)		
1-5	0 (0.0%)	6 (16.7%)		
5-10	0 (0.0%)	5 (13.9%)		0.001*
>10	0 (0.0%)	7 (19.4%)		
Stromal overgrowth				
Absent	51 (57.3%)	1 (2.8%)		0.000*
Mild	21 (23.6%)	9 (25%)		
Moderate	17 (19.1%)	19 (52.8%)		
Severe	0 (0.0%)	7 (19.4%)		
Stromal cellularity adjacent to epithelium				
Absent	59 (66.3%)	20 (55.6%)		0.05
Not Applicable	0 (0.0%)	2 (5.6%)		
Present	30 (33.7%)	14 (38.9%)		

In the FA group, the stromal overgrowth on trucut specimens was absent in 51 (57.3%), mild in 21 (23.6%), and moderate in 17 (19.1%). But, in PT the stromal overgrowth was absent in one (2.8%), mild in nine (25.0%), moderate in 19 (52.8%), and severe in seven (19.4%) patients.

In the FA group, the enhancement of stromal cellularity adjacent to the epithelium was absent in 59 (66.3%) and present in 30 (33.7%). While in PT, it was absent in 20 (55.6%), not applicable to two (5.6%), and present in 14 (38.9%) patients (Table 4).

**Table 4:** Comparison of the stroma to epithelial proportion and the outcome

Stroma to epithelial proportion	Outcome		Total	P-value
	Fibroadenoma at excision	Phyllodes tumor at excision		
30.00%	1 (1.1%)	0 (0.0%)	1 (0.8%)	0.001
40.00%	6 (6.7%)	1 (2.8%)	7 (5.6%)	
50.00%	47 (52.8%)	4 (11.1%)	51 (40.8%)	
60.00%	13 (14.6%)	6 (16.7%)	19 (15.2%)	
70.00%	11 (12.4%)	9 (25.0%)	20 (16.0%)	
80.00%	10 (11.2%)	8 (22.2%)	18 (14.4%)	
90.00%	1 (1.1%)	5 (13.9%)	6 (4.8%)	
95.00%	0 (0.0%)	1 (2.8%)	1 (0.8%)	
99.00%	0 (0.0%)	2 (5.6%)	2 (1.6%)	
Total	89 (100.0%)	36 (100.0%)	125 (100.0%)	

## Discussion

In females, breast tumors are common malignancies and among these, benign lesions are frequent. FAs are one of the common lesions that range from benign to pre-malignant conditions as phyllodes. So, it is of paramount importance to outline the histologic parameters that could be convenient in segregating the FA and phyllodes, even on trucut biopsy samples.

In our study, the mean age was 33.86 ± 9.95 years. It showed that young women are quite common to have such tumors. We found that 36 (28.8%) patients had PT at excisional biopsy who were diagnosed as FA on trucut samples. Al-Arnawoot et al. conducted a study with 134 cellular fibroepithelial lesions (CFEL). In their study, they found that Eighty-nine 89 (66%) were FA and 44 (34%) were phyllodes (32 benign; 13 malignant) [14].

Differentiating PT from FA in the preoperative context is a matter of great importance in clinical practice since the conservative management of patients can be worrisome. In a study by Reis et al., they found that fibroepithelial tumors diagnosed after the trucut biopsy, but having a larger size of more than 30 mm on ultrasound, have a greater probability of being pre-malignant. So, fibroepithelial lesions larger than 30 cm are more likely to be PT and are coherent with the existing medical literature [2]. Significant predictors of increased risk of PT were patient age equal to or greater than 50 years (P = 0.021) and lesion size more than 30 mm at sonography [14]. When we compare the percentage of diagnosis on excisional biopsy with ultrasonographic size, it revealed that the tumors with more than 30 mm in size were frequently phyllodes on excisional biopsy 31 (86%) (p-value 0.000).

**Figure 1:**
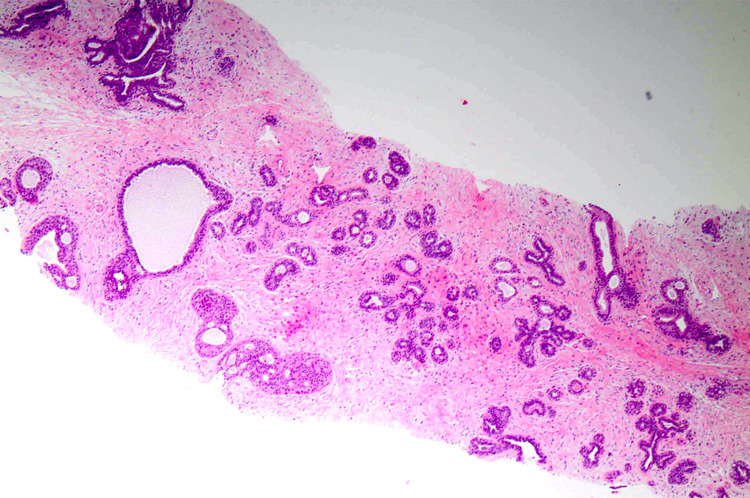
Fibroadenoma on trucut sample showing no atypia and cellularity

**Figure 2:**
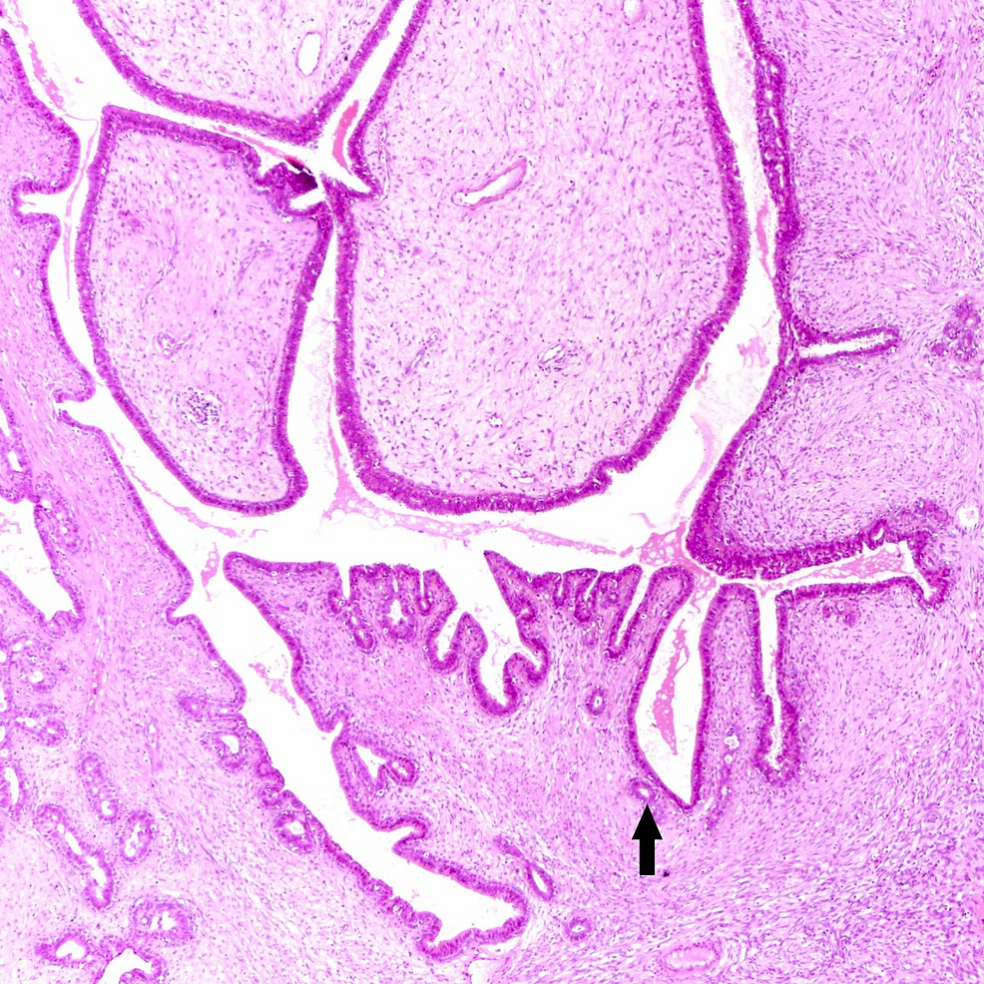
Benign phyllodes showing leaflike growth pattern with no atypia and mild cellularity

**Figure 3:**
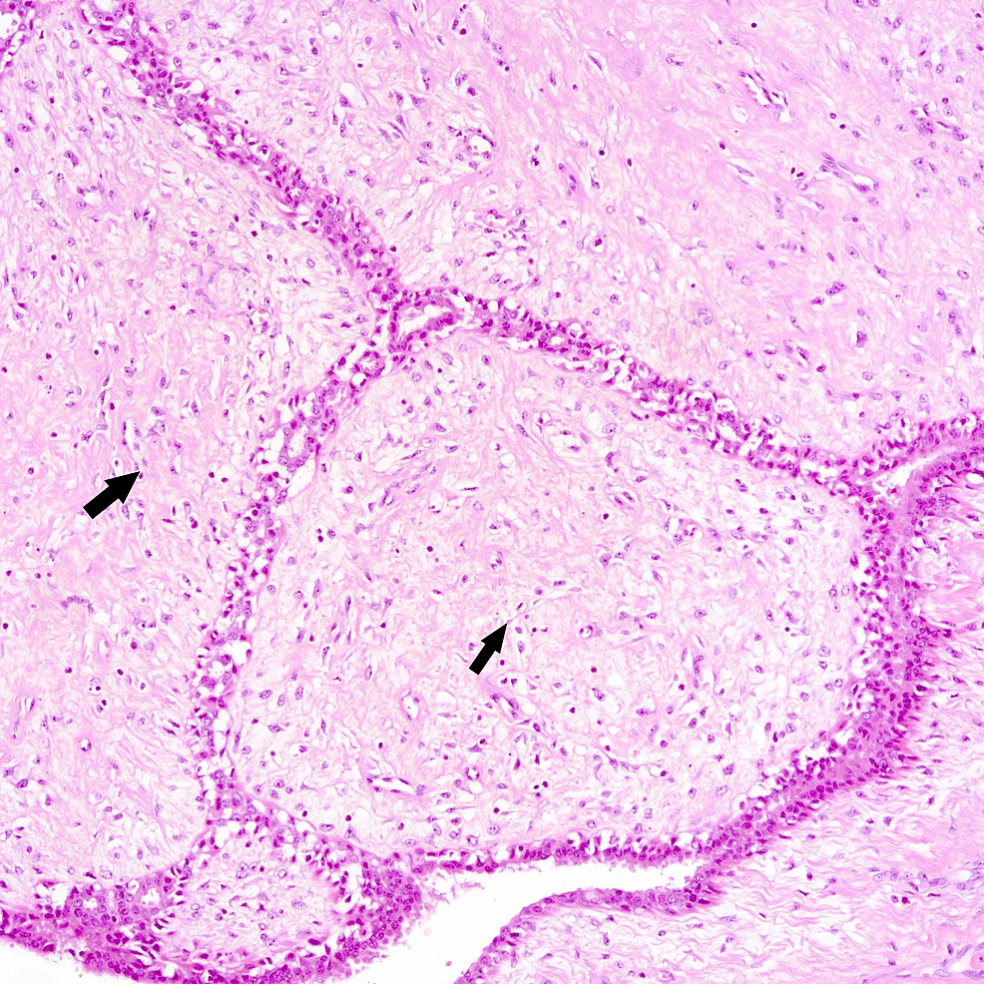
Borderline phyllodes showing mild atypia and stromal cellularity with one to two mitosis 10hpf and no pushing margins (core biopsy)

In our study, the upgrade rate from fibroepithelial lesions to PTs is 48.3%. These included mainly the tumors with a size of more than 30 mm, increased stromal cellularity, and fibroepithelial lesions with spindle cell proliferation. Moreover, our study also highlights that PTs signify severe stromal cellularity 10 (27.8%) (p-value 0.001) and severe stromal overgrowth 07 (19.4%) (p-value 0.000) on trucut biopsy specimens as compared to FAs who had mild to moderates activities of each primarily. These features are necessary to be considered for diagnosing the phyllodes as a marker of surgical excision. Zhou et al. had described the sensitivity of core needle biopsy (CNB) at diagnosing benign, borderline, and malignant PTs as 4.9% (2/41), 4.2% (3/71), and 25.0% (4/16), respectively, that showed poor accuracy as a guide for surgical decisions if considered without these histological features (Figures 1-3) [15].

It is also worth mentioning that there is a difference in trucut biopsy accuracy in diagnosing benign, malignant, and borderline lesions. Ward et al. have conducted a study on 91 phyllodes patients after excision biopsy and concluded that the trucut method was less sensitive alone. However, additional ultrasonography may help to predict the phyllodes more accurately [16].

Our findings recommend that the mitotic count at trucut biopsy is also a valuable indicator for assessing the FA or the phyllodes. So, the specimens with high mitotic activity may better propose as phyllodes but it could not be a solitary marker as still, 50% of patients among phyllodes were having mitotic activity of 0-1 only (p-value 0.001). Likewise, the enhancement of stromal cellularity adjacent to epithelium may reveal a better emblem to assign a patient either to be observed or plan for excision of the tumor (p-value 0.05). In a study by Jacobs et al., stromal cellularity enhancement was observed 33% in the FA group and 63% in the phyllodes group [3].

**Figure 4:**
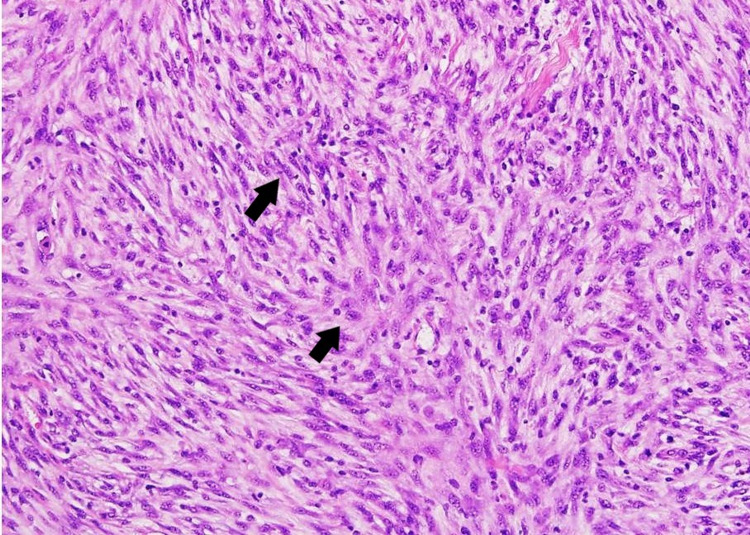
Malignant phyllodes showing marked atypi stromal cellularity and atypical mitosis (excision biopsy)

As in our study, the findings showed that stromal cellularity, atypia, and mitotic count were different in the FA and the phyllodes. Yasir et al. conducted a study and enrolled 64 patients. Twenty-seven (42.2%) of 64 were diagnosed as PT (24 as benign patients and three borderlines) and 37 (57.8%) as cellular FA on excision biopsy. All features except for increased stromal cellularity were statistically significant in that study (Figure 4) [17].

For better-quality management of patients, it is imperative to differentiate between the FA and phyllodes. These tumors have variable courses and outcomes as also found in some other studies [18,19]. Lastly, our results advance the prospect that patients with tumor size < 30 mm, mild stromal cellularity, stomal overgrowth, low mitotic activity, and mild stromal cellularity adjacent to epithelium on trucut biopsy specimens may be observed and followed over time. Stroma to epithelial proportion less than 50% are also found to be associated with fewer chances of phyllodes (p-value 0.001).

There are few confines of our study. It was a retrospective analysis. Trucut biopsy was performed by different personals, rendering a variable chance of histology result on trucut biopsy. Although our results are statistically significant, we need large data to validate our conclusions. Further, there is a need for comparative diagnostic modalities for dual verification of impressions like magnetic resonance imaging. So, in this reverence, we are arranging a multi-institutional prospective study over a large period of time to find the precise fallouts that could be executed over a large scale for better influence.

## Conclusions

Assessment of tumor size, stromal cellularity, mitoses, and enhancement of stromal cellularity adjacent to the epithelium in trucut needle biopsy of breast tumors are useful markers for judging the odds of being an FA or PT. The tumors with a size of more than 30 mm, with increased stromal cellularity, and fibroepithelial lesions with spindle cell proliferation are appropriate indicators for excision.

A combination of indicators is better than a single marker. We suggest that patients with tumor size < 30 mm, mild stromal cellularity, stomal overgrowth, low mitotic activity, and mild stromal cellularity adjacent to epithelium on trucut biopsy specimens may be observed and followed over time.
